# When Lynch syndrome is not MSI-H: embryonal testicular carcinoma treated with combined BEP chemotherapy and immunotherapy despite MSS/low TMB: a case report

**DOI:** 10.3389/fonc.2026.1847167

**Published:** 2026-09-16

**Authors:** Nicole A. Lisek, Justin G. Kusiel, Zin W. Myint

**Affiliations:** 1 Department of Internal Medicine, University of Kentucky, Lexington, KY, United States; 2 Department of Internal Medicine, Division of Medical Oncology, University of Kentucky, Lexington, KY, United States

**Keywords:** embryonal carcinoma, germ cell tumor (GCT), Lynch syndrome, microsatellite stability (MSS), MSH2

## Abstract

Immune checkpoint inhibitors have demonstrated minimal activity in unselected testicular germ cell tumors (GCTs), largely due to low tumor mutational burden (TMB) and immunologically “cold” tumor microenvironments. Microsatellite instability–high (MSI-H) or mismatch repair deficiency (dMMR), key predictors of response to PD-1 blockade, are exceedingly rare in GCTs. In patients with Lynch syndrome, germline mismatch repair mutations do not uniformly translate to tumor-level MSI-H/dMMR, creating therapeutic uncertainty. Case Presentation: We report a 33-year-old male with germline MSH2-associated Lynch syndrome who presented with metastatic pure embryonal testicular carcinoma and markedly elevated tumor markers. Imaging demonstrated extensive hepatic and nodal metastases. Following orchiectomy, he was treated with bleomycin, etoposide, and cisplatin (BEP). Given his high-risk disease and germline mutation, pembrolizumab was added starting cycle 2 after shared decision-making, prior to tumor molecular results. Treatment was complicated by neutropenic fever and cytopenias requiring dose reductions and supportive care but no unexpected immune-related adverse events. Tumor markers rapidly declined, and interval imaging demonstrated near-complete resolution of hepatic metastases and significant nodal response. Subsequent next-generation sequencing revealed microsatellite stable (MSS) disease with low TMB (2 mutations/Mb), without actionable alterations. The patient completed therapy and transitioned to maintenance pembrolizumab. Conclusion: This case highlights the clinical and biologic discordance between germline Lynch syndrome and tumor-level biomarkers in a non-Lynch spectrum malignancy. While combination chemotherapy and immunotherapy was feasible, the observed response is consistent with expected cisplatin sensitivity, and the contribution of pembrolizumab remains unclear. These findings underscore the importance of tumor-specific molecular testing and caution against extrapolating germline status to guide immunotherapy decisions in germ cell tumors.

## Introduction

Embryonal testicular carcinoma represents biologically aggressive subsets of germ cell tumors (GCTs), yet most retain high chemosensitivity and favorable long-term outcomes with cisplatin-based therapy. In contrast, immune checkpoint inhibitors have demonstrated minimal activity in unselected testicular GCTs, largely attributed to low tumor mutational burden (TMB), low programmed death-ligand 1 (PD-L1) expression, and immunologically “cold” tumor microenvironments (2–5). Microsatellite instability–high (MSI-H) or mismatch repair deficiency (dMMR), a well-established predictor of response to programmed cell death protein 1 (PD-1) blockade, is exceedingly rare in testicular cancers and is primarily described in treatment-resistant disease rather than at baseline (1, 6–10). Despite the curability of most GCTs with standard chemotherapy, there is limited guidance for incorporating precision oncology approaches in patients with hereditary cancer syndromes.

Lynch syndrome is an autosomal dominant hereditary cancer predisposition syndrome caused by germline mutations in mismatch repair (MMR) genes, most commonly *MLH1*, *MSH2*, *MSH6*, and *PMS2*. While Lynch syndrome-associated tumors frequently demonstrate MSI-H/dMMR status and robust responses to immunotherapy, this relationship is strongest in colorectal, endometrial, gastric, and select urothelial malignancies (11–16). Importantly, not all tumors arising in Lynch syndrome patients exhibit MSI-H/dMMR biology, particularly outside the classic Lynch tumor spectrum. Germline MMR mutations do not uniformly translate into tumor-level dMMR. Non-Lynch spectrum tumors in patients with germline MSH2 mutations may demonstrate microsatellite stability (MSS) and low TMB, thereby lacking the predictive biomarkers typically associated with response to PD-1 blockade (14). This germline-somatic discordance presents a therapeutic dilemma in precision oncology.

Pembrolizumab was granted tumor-agnostic U.S. Food and Drug Administration (FDA) approval in 2017 for unresectable or metastatic MSI-H/dMMR solid tumors, based on durable responses across diverse malignancies (1). The disconnect between tumor-agnostic regulatory approval and tumor-specific biologic uncertainty poses a clinical dilemma in rare malignancies. However, the application of immunotherapy in Lynch syndrome-associated cancers without confirmed tumor MSI-H/dMMR status remains controversial. We present a rare case of metastatic embryonal testicular carcinoma in a patient with confirmed germline *MSH2* mutation who was treated with combined chemotherapy and immunotherapy, highlighting the clinical reasoning, feasibility, uncertainty, and limitations inherent in germline-driven treatment decisions.

## Case report

A 33-year-old male with germline *MSH2*-associated Lynch syndrome (5′UTR_EX6del) and no prior malignancy presented with back pain and was found to have extensive metastatic disease on computed tomography (CT) at an outside hospital. He was subsequently transferred for tertiary oncologic care. For de-identification purposes, the clinical timeline is presented using relative months, with Month 1 corresponding to the patient’s initial presentation.

Initial CT imaging in Month 1 demonstrated innumerable hepatic metastatic lesions, extensive mesenteric and retroperitoneal lymphadenopathy, and an asymmetrically enlarged left testicle suspicious for a primary testicular neoplasm. A tiny right upper lobe pulmonary nodule was also noted. Laboratory evaluation revealed mildly reduced lymphocytes, elevated liver enzymes (aspartate aminotransferase (AST) 102 U/L, alanine aminotransferase (ALT) 40 U/L, alkaline phosphatase 182 U/L), and hyperbilirubinemia (total bilirubin 3.7 mg/dL). Tumor markers were notable for markedly elevated beta-human chorionic gonadotropin (β-hCG) at 44 521 mIU/mL, elevated lactate dehydrogenase (LDH) of approximately 2000 U/L, and alpha-fetoprotein (AFP) of 7.3 ng/mL (**Table 1**).

**Table 1:** Tumor marker trends.

Treatment phase	β-hCG (mIU/mL)	LDH (U/L)	AFP (ng/mL)	Comments
Pre-Orchiectomy (Pre-Cycle 1)	44 521	2610	7.3	
Post-Orchiectomy (Pre-Cycle 1)	219 701	4540	9.5	Aggressive disease biology, Stage IIIC
Cycle 1	—	1685	—	Cycle 1: delayed for neutropenic fever
Cycle 2 (Start)	4286	561	7.2	Cycle 2: Cis/Etop 20% dose reduction
Cycle 2–3 Interval	—	233	—	Cycle 3: 1-week delay for neutropenia; 20% DR
Cycle 3 End	2.06	—	—	Cycle 4: 1-week delay for ANC/NF; 20% DR; G-CSF added

β-hCG, beta-human chorionic gonadotropin; LDH, lactate dehydrogenase; AFP, alpha-fetoprotein; Cis/Etop, cisplatin and etoposide; DR, dose reduction; ANC, absolute neutrophil count; NF, neutropenic fever; G-CSF, granulocyte colony-stimulating factor.

Magnetic resonance imaging (MRI) of the brain in Month 1 showed no intracranial metastases. The patient underwent left radical orchiectomy shortly after. Pathology revealed a GCT composed of pure embryonal carcinoma. Post-orchiectomy β-hCG levels increased to 219 701 mIU/mL, consistent with aggressive disease biology. The patient was classified as having stage IIIC nonseminomatous GCT.

Standard treatment with bleomycin, etoposide, and cisplatin (BEP) chemotherapy was initiated (**Figure 1**). Given the patient’s known *MSH2* germline mutation associated with Lynch syndrome and his high-risk metastatic presentation, pembrolizumab was added off-label with cycle 2 after shared decision-making. At the time of initiation, next-generation sequencing (NGS) results were not yet available, and thus, tumor MSI/MMR status was not known at the time of immunotherapy initiation. Prior to initiation of therapy, the patient had elevated liver function tests attributed to extensive hepatic metastases (AST 150 U/L, ALT 42 U/L, total bilirubin 2.2 mg/dL, alkaline phosphatase 311 U/L), prompting a 20% dose reduction in etoposide. During cycle 1, he was hospitalized for neutropenic fever (maximum temperature 104°F), pancytopenia (white blood cell count <0.3, hemoglobin 4.3, absolute neutrophil count 0.02) requiring broad-spectrum antibiotics, transfusions, and dental extraction for suspected odontogenic source. Subsequent cycles required continued dose reductions of cisplatin and etoposide (20% reduction each), transfusion support for anemia and thrombocytopenia, and brief treatment delays for absolute neutrophil count recovery. Given recurrent neutropenic fever, granulocyte colony-stimulating factor was added for the final fourth cycle. Despite these complications, no unexpected immune-related adverse events were observed during combination therapy. Specifically, no immune-mediated hepatitis, pneumonitis, endocrinopathy, or other grade ≥3 immune-related adverse events were observed during combination treatment and no overlapping pulmonary toxicity attributable to bleomycin and checkpoint blockade was identified. NGS from radical orchiectomy specimen returned at the end of cycle 4 and demonstrated MSS and low TMB (2 mutations/Mb). No actionable somatic alterations were identified, including mutations in *BRAF*, *KRAS*, *NRAS*, or *KIT*, *ERBB2* amplification, or *NTRK1/2/3* and *RET* gene fusions.

**Figure 1:**
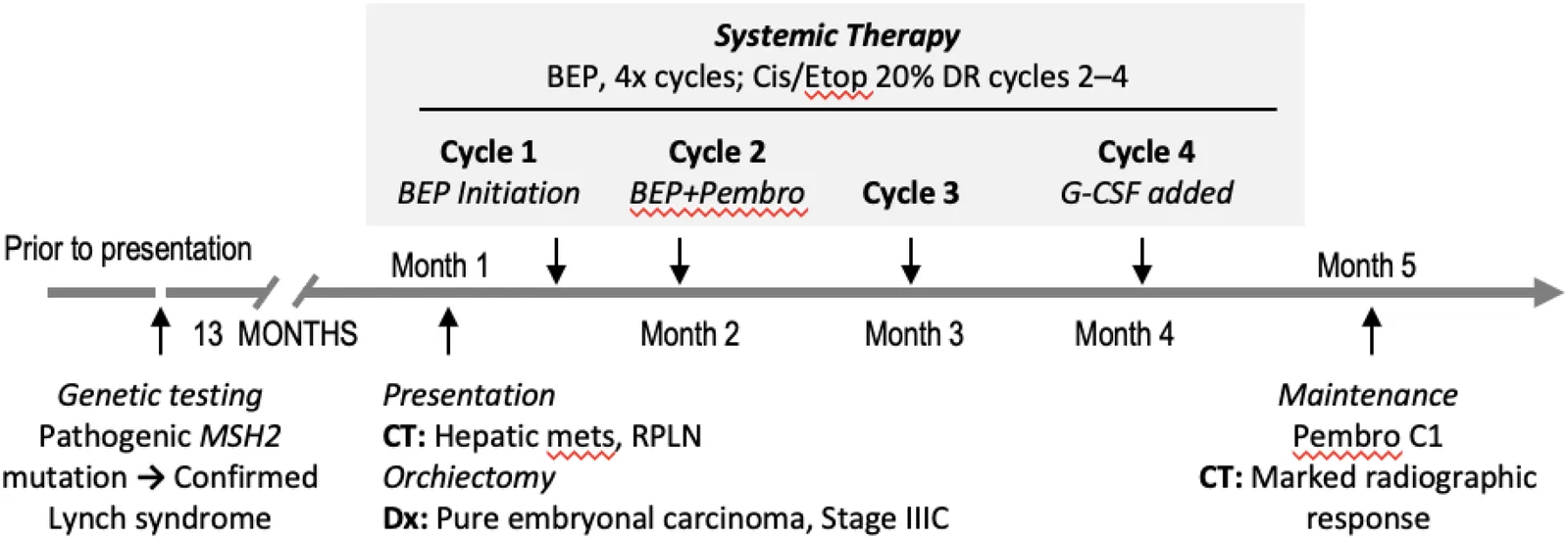
Case timeline. (BEP, bleomycin, etoposide, and cisplatin; Cis/Etop, cisplatin, etoposide; DR, dose reduction; Pembro, pembrolizumab; G-CSF, granulocyte colony-stimulating factor; RPLN, retroperitoneal lymph node).

Tumor markers demonstrated substantial response, with β-hCG declining from 44 521 mIU/mL at baseline to 4286 mIU/mL following initiation of therapy and to 2.06 mIU/mL by completion of cycle 3. Follow-up CT imaging at the end of Month 5 showed near complete resolution of hepatic metastatic burden (with only a few small low-attenuation foci noted) and decreased size of mediastinal and retroperitoneal lymphadenopathy, consistent with treatment response (**Figure 2**). Pulmonary nodules remained stable.

**Figure 2:**
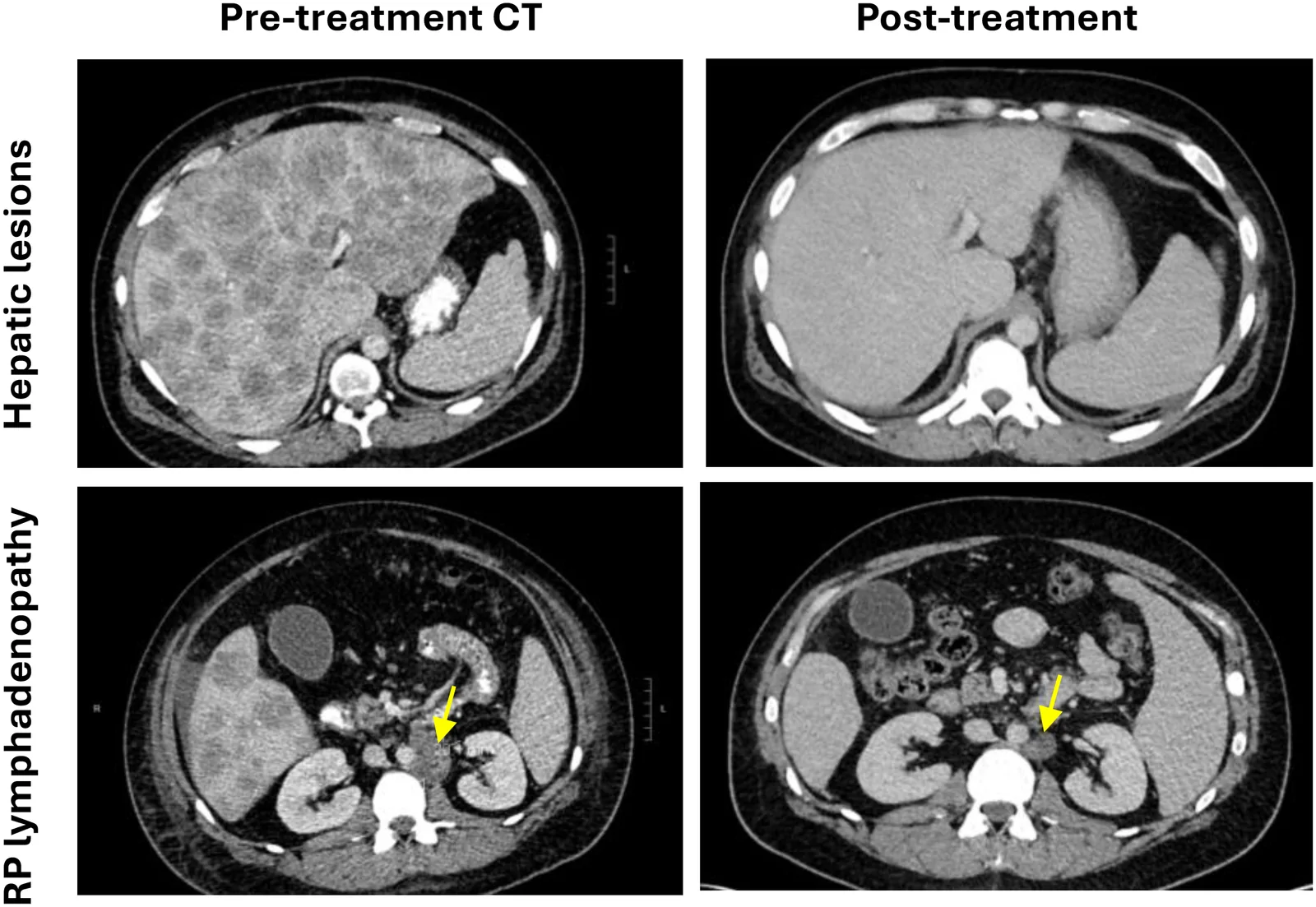
Contrast-enhanced CT images demonstrating hepatic metastases and retroperitoneal lymphadenopathy at diagnosis (Month 1) and after completion of combination therapy (Month 5) showing significant radiographic response.

Following completion of combination therapy, the patient initially transitioned to maintenance pembrolizumab following multidisciplinary discussion, recognizing the absence of tumor-level MSI-H/dMMR biomarkers and the exploratory nature of continued immune checkpoint blockade. Once NGS from the orchiectomy specimen returned demonstrating MSS and low TMB, maintenance pembrolizumab was discontinued. As of Month 12, the patient remains clinically stable with no evidence of disease progression.

## Discussion

This case illustrates the complex intersection of hereditary cancer predisposition and real-world decision-making in the absence of tumor-specific biomarkers. Pembrolizumab received tumor-agnostic FDA approval in 2017 for unresectable or metastatic MSI-H/dMMR solid tumors. However, germline Lynch syndrome does not uniformly predict tumor MSI-H/dMMR status, particularly outside the classic Lynch tumor spectrum (1). Although Lynch syndrome-associated tumors frequently demonstrate MSI-H biology, this association is strongest within the classic Lynch tumor spectrum and is significantly less consistent in non-colorectal, non-endometrial malignancies (14, 15). In a prospective cohort of 75 patients with Lynch syndrome-associated colorectal cancer, Yu et al. reported an objective response rate of 70.7%, including complete responses in 28% of patients, with a 3-year overall survival of 91.2%. Importantly, responses were restricted to tumors with confirmed dMMR/MSI-H status (16). Notably, the tumor-agnostic approval of pembrolizumab requires demonstration of MSI-H or dMMR within the tumor itself rather than germline mutation alone. In this case, tumor sequencing demonstrated MSS and low TMB, consistent with germline-tumor discordance between the patient’s known pathogenic MSH2 variant and tumor phenotype.

Lynch syndrome affects approximately 1 in 300 individuals, with *MSH2* mutations accounting for a substantial proportion of extracolonic malignancies. Testicular GCTs are not considered part of the classic Lynch tumor spectrum, and the coexistence of germline *MSH2*-associated Lynch syndrome with pure embryonal carcinoma is exceedingly rare. Pure embryonal carcinoma itself represents an aggressive subtype of nonseminomatous GCT characterized by early hematogenous dissemination and marked tumor marker elevation. Our patient presented with stage IIIC disease, including extensive hepatic metastases and markedly elevated post-orchiectomy β-hCG levels, placing him in a poor-risk category. Although cisplatin-based chemotherapy remains curative for many advanced GCTs, the presence of non-pulmonary visceral metastases significantly reduces the probability of durable cure. The substantial biochemical and radiographic response observed is consistent with expected outcomes from cisplatin-based chemotherapy in advanced GCTs.

GCTs represent a particularly challenging context for immunotherapy. Multiple prospective studies have demonstrated minimal activity of pembrolizumab in unselected or platinum-refractory testicular GCTs, with no objective responses reported (2, 3). MSI-H/dMMR status is exceedingly rare in testicular cancer and, when present, is typically associated with cisplatin resistance rather than treatment-naïve disease (6–10). To date, only a single published case report describes a dramatic response to pembrolizumab in MSI-H testicular cancer, confirmed on metastatic tissue despite MSI-negative primary tumor findings (17). To our knowledge, published experience of combined BEP plus pembrolizumab in Lynch syndrome-associated embryonal carcinoma is extremely limited.

The biologic rationale for combining chemotherapy with immunotherapy is supported by data from other malignancies. Cytotoxic chemotherapy can enhance antigen release, promote immune priming, and alter the tumor microenvironment in ways that may augment checkpoint blockade activity. In MSI-H metastatic colorectal cancer, the phase III KEYNOTE-177 trial demonstrated superior progression-free survival with pembrolizumab compared with chemotherapy (18). Similarly, immune checkpoint blockade has shown significant efficacy in dMMR endometrial cancer (KEYNOTE-868) (19). The foundational tumor-agnostic approval of pembrolizumab for MSI-H/dMMR solid tumors further supports the mechanistic plausibility of immune checkpoint inhibition in dMMR tumors (20). While direct evidence in GCTs is lacking, tumor profiling later demonstrated MSS/low TMB, the presence of a pathogenic *MSH2* mutation initially provided biologic plausibility for considering immune modulation in this high-risk setting. This case underscores that germline testing and tumor profiling are complementary rather than interchangeable tools in precision oncology.

Importantly, not all tumors arising in Lynch syndrome patients are MSI-H or characterized by high TMB, and germline genotype is not a reliable surrogate for tumor-level MMR status. In a large multicenter cohort study of patients with germline Lynch syndrome, only 37% of non-colorectal, non-endometrial malignancies exhibited MSI-H/dMMR features compared to 85% of Lynch-spectrum tumors (14). This discordance also occurs within the classic colorectal spectrum. Khandakar et al. described two patients with confirmed Lynch syndrome (germline MSH6 and PMS2 variants) who developed MMR-proficient MSS/pMMR colorectal adenocarcinomas. In one patient with two discordant tumors, a dMMR-lesion achieved a complete pathologic response to pembrolizumab, whereas the MSS/pMMR lesion was poorly responsive (21). Consistent with this, Ranganathan et al. found that 10% of colorectal cancers in Lynch syndrome patients were MSS/pMMR, disproportionately seen in MSH6/PMS2 carriers and more often later-onset and metastatic (22). Discordance between dMMR and TMB has likewise been reported, and tumors with dMMR but low TMB may fail to respond to immune checkpoint inhibition (13). These findings underscore the necessity of tumor-specific testing whenever possible.

Beyond MSI and TMB, response to checkpoint blockade is increasingly understood to depend on additional determinants that may still be occurring in MSS/low-TMB tumors, such as neoantigen quality, antigen-presentation, PD-L1 expression, or the tumor immune microenvironments. The absence of internationally standardized and prospectively validated immunological biomarkers, including PD-L1, TMB, and MSI-H/dMMR, continues to limit treatment selection in cases such as this.

In this case, tumor profiling of the orchiectomy specimen demonstrated MSS and low TMB, highlighting germline discordance. The absence of tumor-level MSI-H/dMMR status weakens the biologic rationale for immune checkpoint inhibition and underscores that germline Lynch syndrome should not be used as a surrogate for tumor MSI-H/dMMR status (13, 14). The substantial clinical response observed in this case is consistent with the known efficacy of cisplatin-based BEP chemotherapy in advanced GCTs, and the independent contribution of pembrolizumab cannot be determined. Moreover, this limited follow-up does not allow the role of maintenance immunotherapy to be verified in either direction. Continued long-term surveillance is warranted given the potential for relapse in poor-risk nonseminonmatous disease. Accordingly, the addition of pembrolizumab was not biomarker-driven but rather an individualized therapeutic decision made in the context of high-risk metastatic disease and shared decision-making. This approach should not be interpreted as endorsement of routine immunotherapy use in GCTs but rather as a hypothesis-generating example of precision oncology decision-making under biologic uncertainty.

From a safety perspective, the combination of BEP and pembrolizumab was generally feasible. The patient experienced neutropenia requiring dose reduction of cisplatin and etoposide and brief treatment delay, toxicities consistent with standard BEP therapy. No unexpected immune-related adverse events were observed during combination therapy, and maintenance pembrolizumab has thus far been tolerated. Given the curative potential of cisplatin-based chemotherapy in GCTs, careful consideration of additive toxicity is essential before adopting such an approach outside individualized cases. Feasibility also varies across health systems. The substantial cost of pembrolizumab and country-specific prescribing and reimbursement restrictions mean access is neither uniform nor guaranteed. This reinforces the value of tumor-specific MSI/MMR testing to ensure that a costly, access-limited therapy is directed only to patients most likely to benefit.

## Conclusion

This case highlights the challenges of applying tumor-agnostic immunotherapy in rare malignancies arising in the context of hereditary cancer syndromes. In a patient with germline *MSH2*-associated Lynch syndrome but tumor MSS/low TMB and high-risk metastatic pure embryonal testicular carcinoma, the addition of pembrolizumab to standard chemotherapy was feasible but not biomarker-supported at the tumor level. While the observed response cannot be attributed to immunotherapy, this report underscores the importance of tumor-specific testing and careful interpretation of germline findings when making precision oncology treatment decisions.

## Data Availability

The original contributions presented in the study are included in the article/supplementary material. Further inquiries can be directed to the corresponding author.
